# Interictal epileptiform discharges as a predictive biomarker for recurrence of poststroke epilepsy

**DOI:** 10.1093/braincomms/fcac312

**Published:** 2022-11-26

**Authors:** Soichiro Abe, Tomotaka Tanaka, Kazuki Fukuma, Soichiro Matsubara, Rie Motoyama, Masahiro Mizobuchi, Hajime Yoshimura, Takayuki Matsuki, Yasuhiro Manabe, Junichiro Suzuki, Hiroyuki Ishiyama, Maya Tojima, Katsuya Kobayashi, Akihiro Shimotake, Kunihiro Nishimura, Masatoshi Koga, Kazunori Toyoda, Shigeo Murayama, Riki Matsumoto, Ryosuke Takahashi, Akio Ikeda, Masafumi Ihara, PROPOSE Study Investigators, Kazuyuki Nagatsuka, Fumiaki Nakamura, Shinya Tomari, Yoshitaka Yamaguchi, Takashi Nakamura, Naoki Makita, Yuki Nakamura, Yoshiaki Okuno, Satoshi Hosoki, Ryo Fujii, Takuro Arimizu

**Affiliations:** Department of Neurology, National Cerebral and Cardiovascular Center, Osaka 5648565, Japan; Department of Neurology, National Cerebral and Cardiovascular Center, Osaka 5648565, Japan; Department of Neurology, National Cerebral and Cardiovascular Center, Osaka 5648565, Japan; Department of Neurology, Graduate School of Medical Sciences, Kumamoto University, Kumamoto 8608556, Japan; Department of Neurology, Tokyo Metropolitan Geriatric Hospital and Institute of Gerontology, Tokyo 1730015, Japan; Department of Neurology, Nakamura Memorial Hospital, Sapporo 0608570, Japan; Clinic of Minami-ichijyo Neurology, Sapporo 0600061, Japan; Department of Neurology, Kobe City Medical Center General Hospital, Kobe 6500047, Japan; Department of Neurology, St Mary’s Hospital, Fukuoka 8300047, Japan; Department of Neurology, National Hospital Organization Okayama Medical Center, Okayama 7011192, Japan; Department of Neurology, Toyota Memorial Hospital, Toyota 4718513, Japan; Department of Neurology, National Cerebral and Cardiovascular Center, Osaka 5648565, Japan; Department of Neurology, Kyoto University Graduate School of Medicine, Kyoto 6068507, Japan; Department of Neurology, Kyoto University Graduate School of Medicine, Kyoto 6068507, Japan; Department of Neurology, Kyoto University Graduate School of Medicine, Kyoto 6068507, Japan; Department of Preventive Medicine and Epidemiology, National Cerebral and Cardiovascular Center, Osaka 5648565, Japan; Department of Cerebrovascular Medicine, National Cerebral and Cardiovascular Center, Osaka 5648565, Japan; Department of Cerebrovascular Medicine, National Cerebral and Cardiovascular Center, Osaka 5648565, Japan; Department of Neurology, Tokyo Metropolitan Geriatric Hospital and Institute of Gerontology, Tokyo 1730015, Japan; Division of Neurology, Kobe University Graduate School of Medicine, Kobe 6500017, Japan; Department of Neurology, Kyoto University Graduate School of Medicine, Kyoto 6068507, Japan; Department of Epilepsy, Movement Disorders and Physiology, Kyoto University Graduate School of Medicine, Kyoto 6068507, Japan; Department of Neurology, National Cerebral and Cardiovascular Center, Osaka 5648565, Japan

**Keywords:** interictal epileptiform discharge, periodic discharge, poststroke epilepsy, seizure recurrence

## Abstract

Poststroke epilepsy is a major ischaemic/haemorrhagic stroke complication. Seizure recurrence risk estimation and early therapeutic intervention are critical, given the association of poststroke epilepsy with worse functional outcomes, quality of life and greater mortality. Several studies have reported risk factors for seizure recurrence; however, in poststroke epilepsy, the role of EEG in predicting the risk of seizures remains unclear. This multicentre observational study aimed to clarify whether EEG findings constitute a risk factor for seizure recurrence in patients with poststroke epilepsy. Patients with poststroke epilepsy were recruited from the PROgnosis of POst-Stroke Epilepsy study, an observational multicentre cohort study. The enrolled patients with poststroke epilepsy were those admitted at selected hospitals between November 2014 and June 2017. All patients underwent EEG during the interictal period during admission to each hospital and were monitored for seizure recurrence over 1 year. Board-certified neurologists or epileptologists evaluated all EEG findings. We investigated the relationship between EEG findings and seizure recurrence. Among 187 patients with poststroke epilepsy (65 were women with a median age of 75 years) admitted to the lead hospital, 48 (25.7%) had interictal epileptiform discharges on EEG. During the follow-up period (median, 397 days; interquartile range, 337–450 days), interictal epileptiform discharges were positively correlated with seizure recurrence (hazard ratio, 3.82; 95% confidence interval, 2.09–6.97; *P* < 0.01). The correlation remained significant even after adjusting for age, sex, severity of stroke, type of stroke and generation of antiseizure medications. We detected periodic discharges in 39 patients (20.9%), and spiky/sharp periodic discharges were marginally associated with seizure recurrence (hazard ratio, 1.85; 95% confidence interval, 0.93–3.69; *P* = 0.08). Analysis of a validation cohort comprising 187 patients with poststroke epilepsy from seven other hospitals corroborated the association between interictal epileptiform discharges and seizure recurrence. We verified that interictal epileptiform discharges are a risk factor for seizure recurrence in patients with poststroke epilepsy. Routine EEG may facilitate the estimation of seizure recurrence risk and the development of therapeutic regimens for poststroke epilepsy.

## Graphical Abstract

**Graphical Abstract:**
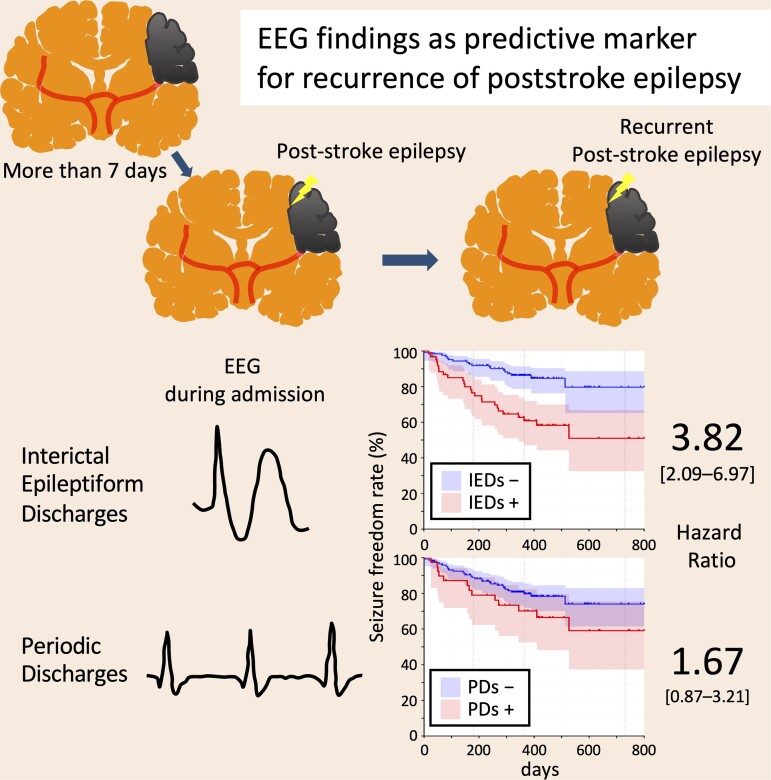

## Teaser

Abe *et al*. report that interictal epileptiform discharges are a risk factor for seizure recurrence in patients with poststroke epilepsy. Routine EEG may facilitate the estimation of seizure recurrence risk and the development of therapeutic regimens for poststroke epilepsy.

## Introduction

Poststroke epilepsy (PSE) is a common complication of stroke. Recent studies have reported that PSE occurs in 10–12% of patients during the 5- to 10-year period after stroke onset.^1,2^ Although antiseizure medications (ASMs) are effective for decreasing seizure recurrence risk, patients with PSE have a higher recurrence even with ASM use. Reports indicate that the cumulative seizure recurrence rates are 42–81 and 28–84% for ischaemic and haemorrhagic stroke, respectively.^3^ It was previously demonstrated that 30% of patients with PSE experienced seizure recurrence during 1-year post discharge.^4^ Another study reported that 20% of patients with PSE were refractory to ASMs.^5^ The International League Against Epilepsy (ILAE) guidelines in 2014 recommended that therapeutic intervention be considered after the first unprovoked seizure >1-week post stroke unless the classical definition of epilepsy was satisfied owing to high seizure recurrence risk.^6,7^ Previous studies have demonstrated that the presence of PSE was associated with neurological worsening,^8^ changing the modified Rankin scale (mRS) from 3 to 4,^9^ and functional decline.^10^ Another study reported that health-related quality of life score was reduced in patients with PSE, and seizure frequency was an independent determinant of health-related quality of life score in patients with PSE.^11^ Furthermore, patients with PSE have a higher risk of short- and long-term mortality within 30-day and 20-year post seizures, respectively.^12^ Therefore, identifying prognostic factors that may facilitate therapeutic intervention or dosage adjustment of ASMs is essential to improve the management of patients with PSE.^13,14^

The risk factors for late poststroke seizure after stroke, such as stroke severity, late seizure, presence and volume of haemorrhagic stroke, younger age and presence of convulsions, remain controversial.^1,4,15–19^ Risk models of late seizure after stroke in patients with ischaemic stroke^20^ and intracerebral haemorrhage have been reported.^21^ EEG is an indispensable tool for diagnosing epilepsy, detecting the epileptogenic zone, identifying non-convulsive status epilepticus and predicting seizure recurrence.^22^ Accordingly, this approach may be useful in PSE management. Nevertheless, there is a paucity of reports investigating the relationship between EEG findings and seizure recurrence, especially in late poststroke seizure,^16,23^ because the detection rate of epileptiform activity is lower in patients with a first unprovoked seizure (12–50%) than in children with epilepsy (18–56%).^24^ This study hypothesized that epileptic discharges on EEG could predict seizure recurrence of PSE. Therefore, this study aimed to assess EEG abnormalities and seizure recurrence in patients with PSE.

## Materials and methods

### Study design and population

This study was conducted as a subanalysis of the multicentre prospective cohort PROgnosis of POst-Stroke Epilepsy (PROPOSE) study (UMIN clinical trial registry: UMIN000019940). This study enrolled consecutive patients with PSE admitted to the cerebrovascular medicine and neurology departments of the National Cerebral and Cardiovascular Center (NCVC) and seven hospitals specializing in stroke and epilepsy care in Japan between November 2014 and June 2017. Based on the new clinical definition according to ILAE,^6^ PSE was diagnosed in individuals aged >20 years with late seizures that occurred >1-week poststroke onset. Individuals were carefully screened to confirm PSE diagnosis using semiology, CT, MRI, single-photon emission computed tomography and EEG. This PROPOSE study excluded patients with (i) acute symptomatic seizures that occurred within 1-week poststroke onset; (ii) a history of asymptomatic stroke; (iii) a history of epilepsy pre-stroke onset; or (iv) other causes of epilepsy or potentially epileptogenic comorbidities such as intracranial tumours, traumatic brain injury, alcohol or drug abuse or other possible causes. Besides the exclusion criteria of the PROPOSE study, patients who did not undergo EEG at admission were also excluded from the present study. The final diagnosis of PSE was verified by certified neurologists and epileptologists at each hospital.

### Standard protocol approvals, registrations and patient consents

Our institutional ethical committee (M26-09-7) and each participating institutional review board approved this study, which was conducted according to the relevant institutional guidelines. The need for informed consent was waived by the ethical committees in accordance with the ‘opt-out’ principle.

### Acquisition of baseline clinical data

Baseline clinical data during hospitalization were systematically acquired, including age, sex, family history of epilepsy, current alcohol consumption, comorbidities (hypertension, hyperlipidaemia and diabetes mellitus), ictal/postictal symptoms, severity of stroke, stroke type, location and size of stroke lesion, history of early/late seizure and ASMs before EEG. Detailed seizure information of the ictal/postictal phase (convulsion, paresis, aphasia and consciousness alternation) from patients and eyewitnesses were collected. Stroke severity using the National Institutes of Health Stroke Scale (NIHSS) was evaluated at the latest stroke onset. Stroke subtypes were categorized as ischaemic stroke, intracerebral haemorrhage or subarachnoid haemorrhage. Haemorrhagic stroke was defined as intracerebral and subarachnoid haemorrhage. We also investigated cortical lesions and stroke size in the largest diameter (categorized as <15, 15–30 and >30 mm) using CT or MRI. The history of early seizures (i.e. occurring within 7 days post stroke) and late seizures (i.e. occurring >7-days post stroke) was assessed. At discharge, data on oral ASMs for secondary prevention into older- (valproic acid, carbamazepine, phenytoin, clonazepam and phenobarbital) or newer generations (levetiracetam, lamotrigine, lacosamide, zonisamide, gabapentin and perampanel) were collected and classified. Patients were treated with ASMs at the discretion of the attending physicians in accordance with standard clinical practice at each hospital. Functional disability at discharge using the mRS was assessed.

### EEG findings

After admission, standard scalp EEG was performed for ∼20 min based on the international 10–20 system with 21 electrodes using a multichannel EEG machine (Nihon Kohden, Tokyo, Japan). The sampling rate and bandpass filter were set to 500 and 0.53–120 Hz, respectively. Board-certified epileptologists or neurologists, including two epileptologists (K.F., K.K., A.S. and M.T.) at NCVC, an epileptologist (S.M., R.M. and H.Y.) at three other hospitals and a neurologist (T.M., Y.M., M.M. and J.S.) at four hospitals, interpreted EEG results in a manner blinded to other clinical and imaging data. If necessary, EEG was repeated. The following EEG findings were assessed: interictal epileptiform discharges (IEDs), periodic discharges (PDs), rhythmic delta-wave activity (RDA) and focal irregular slow.

According to the International Federation of Clinical Neurophysiology, IEDs were defined as di- or tri-phasic waves with sharp or spiky morphology distinct to background activity, typically followed by an associated slow after wave.^25^ IED morphology (categorized as spike/spike and waves or sharp/sharp and wave), prevalence (categorized as ≥1 per 10 s, ≥1 per 1 min but <1 per 10 s, ≥1 per 5 min but <1 per min or <1 per 5 min) and distribution (categorized as generalized, hemispheric or focal) were also evaluated. PDs were defined as the repetition of a relatively uniform waveform (including spike, sharp, sharply contoured or blunt) with regular intervals, lasting ≤0.5 s and continuing for >6 cycles, according to the American Clinical Neurophysiology Society (ACNS) Standardized Critical Care EEG Terminology.^26^ Furthermore, PDs sharpness, frequency (categorized as >2.5 Hz, >1 Hz but ≤2.5 Hz or ≤1 Hz) and distribution (categorized as generalized, hemispheric or focal) were evaluated. RDA was defined as the repetition of a relatively uniform delta waveform without intervals, with a frequency of 0.5–4 Hz and continuing for >6 cycles, according to the ACNS terminology.^26^ Focal irregular slow activity was defined as focal irregular (excluding rhythmic waves) theta or delta activities observed at a focal area of the scalp region regardless of continuous or intermittent occurrence.

### Assessment of the relationship between EEG findings and seizure recurrence

In accordance with standard clinical practice at each hospital, patients enrolled in PSE were followed up for at least 1 year after discharge. Board-certified epileptologists or neurologists performed follow-ups at 6 and 12 months after discharge at the outpatient clinic and assessed episodes of unprovoked seizure recurrences. If outpatient visits were not feasible, telephone interviews with patients, relatives or primary care physicians were conducted 6 and 12 months after discharge. The relationship between EEG findings (IEDs, PDs, RDA and focal irregular slow) and seizure recurrence was evaluated.

### Validation

The outcomes of seizure recurrence were collected from the PROPOSE study.

We used data from seven hospitals other than the NCVC as a validation cohort to verify the association between IEDs and seizure recurrence. This validation cohort had the same study period and inclusion/exclusion criteria as the NCVC cohort. The investigators at each hospital confirmed the presence of IEDs based on the same definition as that of NCVC.

### Statistical analysis

Continuous values are presented as median [interquartile range (IQR)], whereas categorical variables are expressed as counts (percentages). Wilcoxon rank-sum test was used to analyse continuous variables and Pearson’s *χ*^2^ test or Fisher’s exact test to analyse categorical variables. We compared EEG findings and seizure recurrence using Cox proportional hazards models and calculated the hazard ratio (HR) with 95% confidence interval (CI) using univariable and multivariable regression analyses adjusted for potential risk factors for seizure recurrence (Model 1: age, sex and NIHSS; Model 2: age, sex, NIHSS, haemorrhagic stroke and newer-generation ASMs at discharge).^1,4,15–19^ The follow-up time was defined as the period from the discharge date from PSE to the date of death, loss or the final follow-up visit within 800 days. Seizure freedom rates between patients with and without IEDs and those with and without PDs were compared using Kaplan–Meier analysis and the log-rank test, respectively. For sensitivity analyses, the HR of IEDs for seizure recurrence in subgroups stratified by age, sex, convulsion symptoms, stroke type, location and size of stroke lesion and ASMs before EEG or at discharge was illustrated in a forest plot. The association of detailed EEG findings on IEDs and PDs with seizure recurrence was investigated using a univariate Cox proportional hazard model. In the validation analysis, the time-to-event between IEDs and no IEDs was compared using a log-rank test. Statistical significance was defined as a two-sided *P*-value <0.05. Statistical analyses were performed using JMP 15 software (SAS Institute Inc, Cary, NC, USA).

## Results

**Figure 1:**
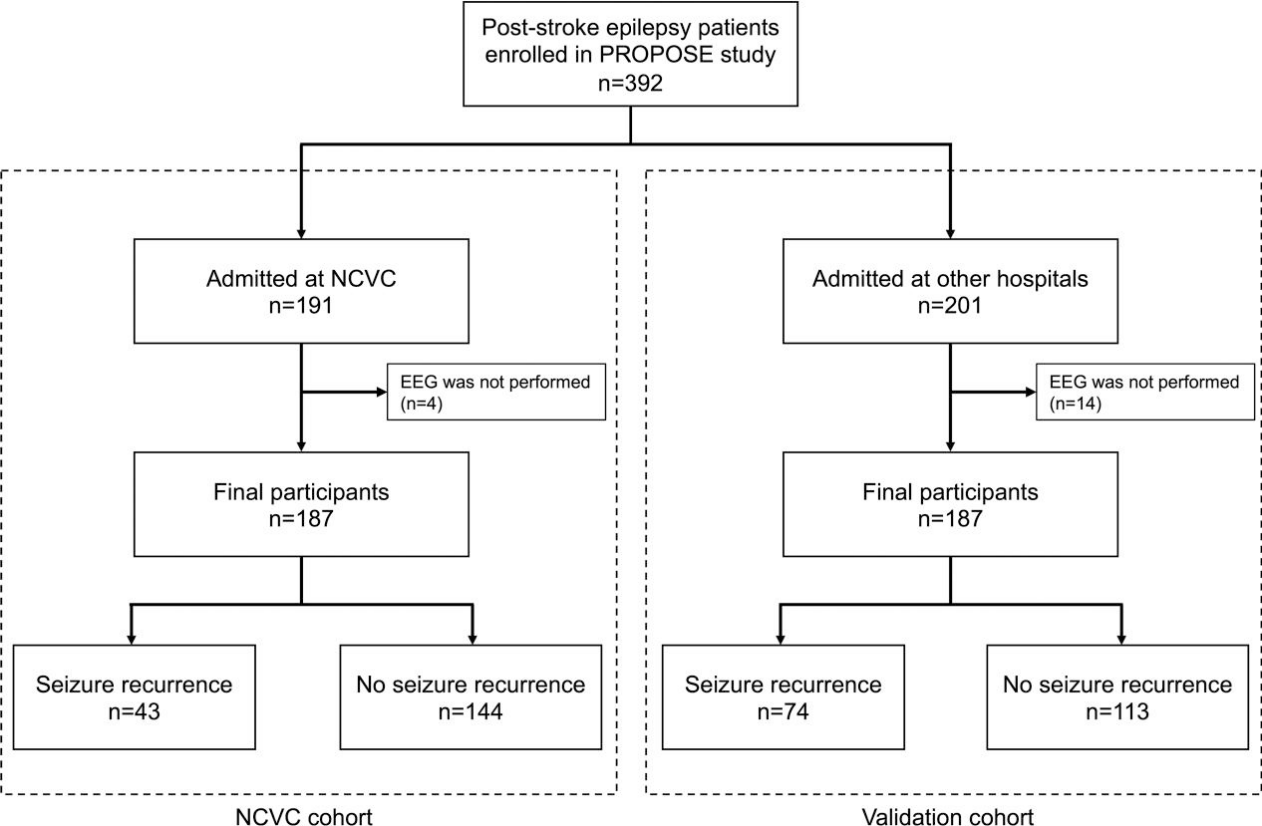
**Study flow chart.** A total of 392 participants were enrolled in the PROPOSE study. In the NCVC cohort, 187 participants were recruited after exclusion. In the validation cohort, 187 participants were recruited from 7 hospitals other than the NCVC.

A total of 191 patients diagnosed with PSE at the NCVC were enrolled between November 2014 and September 2018 (Fig. 1). The final participants were 187 (65 women, 34.8%; median age, 75 years; IQR, 66–82 years), excluding 4 who did not undergo EEG at admission. In total, 28 patients (15.0%) dropped out within 1 year because of death (*n* = 24) or difficulty of contact (*n* = 4). During the follow-up period (median, 397 days; IQR, 337–450 days), 43 (23.0%) of 187 patients with PSE experienced seizure recurrence. Patients with seizure recurrence had higher NIHSS and higher use of newer-generation ASMs, whereas there was no difference between classifications of seizure (Supplementary Table 1).

Table 1 presents the baseline characteristics of patients with or without IEDs at NCVC. Patients with IEDs (IEDs group) were more likely to be women (29.5 versus 50.0%, *P* = 0.01). There were no significant differences in comorbidities or ictal/postictal symptoms. Regarding stroke characteristics, the IEDs group had higher NIHSS [11 (4–19) versus 19 (11–25.5), *P* = 0.02] and higher proportions of intracerebral haemorrhage (56.3%, *P* = 0.006) and haemorrhagic stroke (60.4%, *P* = 0.04). The proportions of cortical and large stroke lesions were not significantly different. Patients receiving levetiracetam monotherapy were more common in the non-IED group than in the IED group (73.0 versus 55.3%, *P* = 0.03). The details of ASM regimens are described in Supplementary Table 2.

**Table 1:** Patient characteristics at baseline

	Non-IEDs	IEDs	*P*-value
	(*n* = 139)	(*n* = 48)	
Age, years, median (IQR)	72.5 (64–82.3)	75 (68–82)	0.20
Female (%)	41 (29.5)	24 (50.0)	0.01
Family history of epilepsy	2 (1.4)	1 (2.1)	>0.99
Current alcohol consumption (%)	26 (18.7)	4 (8.3)	0.11
Duration between seizure and EEG findings, days, median (IQR)	1 (1–2.8)	1 (1–2)	0.32
Comorbidities			
Hypertension (%)	117 (84.2)	40 (83.3)	0.89
Hyperlipidaemia (%)	83 (59.7)	24 (50.0)	0.24
Diabetes mellitus (%)	32 (23.0)	8 (16.7)	0.35
Classification of seizures			0.12
Focal aware seizure	25 (18.0)	2 (4.2)	
Focal impaired aware seizure	50 (36.0)	18 (37.5)	
Focal-to-bilateral tonic-clonic seizure	62 (44.6)	27 (56.3)	
Others	2 (1.4)	1 (2.1)	
Ictal/postictal symptom			
Convulsion (%)	92 (66.2)	34 (70.8)	0.55
Ictal/postictal paresis (%)^a^	30 (21.9)	7 (14.9)	0.30
Aphasia (%)^a^	19 (13.9)	11 (23.4)	0.13
Consciousness alternation (%)^a^	56 (40.9)	25 (53.2)	0.14
Clinical/imaging findings of stroke			
NIHSS at the latest stroke onset (IQR)	11 (4–19)	19 (11.5–25.5)	0.02
Stroke type			
Ischaemic stroke (%)	84 (60.4)	22 (45.8)	0.08
Haemorrhagic stroke^b^ (%)	60 (43.2)	29 (60.4)	0.04
Intracerebral haemorrhage (%)	47 (33.8)	27 (56.3)	0.006
Subarachnoid haemorrhage (%)	13 (9.4)	2 (4.2)	0.36
Cortical involvement	116 (83.5)	38 (79.2)	0.50
Stroke lesion size (*n* = 183)			0.89
<1.5 mm (%)	13 (9.6)	4 (8.3)	
15–30 mm (%)	27 (20.0)	11 (22.9)	
>30 mm (%)	95 (70.4)	33 (68.8)	
History of early seizure (%)^c^	8 (7.4)	0 (0)	0.11
History of late seizure (%)^c^	6 (5.6)	2 (5.4)	>0.99
ASMs			
ASMs before EEG (%)	32 (23.0)	5 (10.4)	0.06
ASMs at discharge (%)	126 (90.7)	47 (97.9)	0.12
Newer-generation ASMs^d^	119 (94.4)	41 (87.2)	0.11
Levetiracetam monotherapy	92 (73.0)	26 (55.3)	0.03
Only older-generation ASMs	7 (5.6)	6 (2.8)	0.11
mRS at discharge, median (IQR)	3 (2.3–4)	3 (1–4)	0.10

Data are presented as *n* (%) or median (interquartile range). ASMs, antiseizure medications; IEDs, interictal epileptiform discharges; IQR, interquartile range; mRS, modified Rankin scale; NIHSS, National Institutes of Health Stroke Scale.

^a^ There were missing data in 3 cases, and we analysed 184 cases.

^b^ Haemorrhagic stroke includes cerebral haemorrhage and subarachnoid haemorrhage.

^c^ There were missing data in 32 cases, and we analysed 155 cases.

^d^ Newer-generation ASMs include newer-generation ASMs and combination therapy of newer-generation and older-generation ASMs.

**Figure 2:**
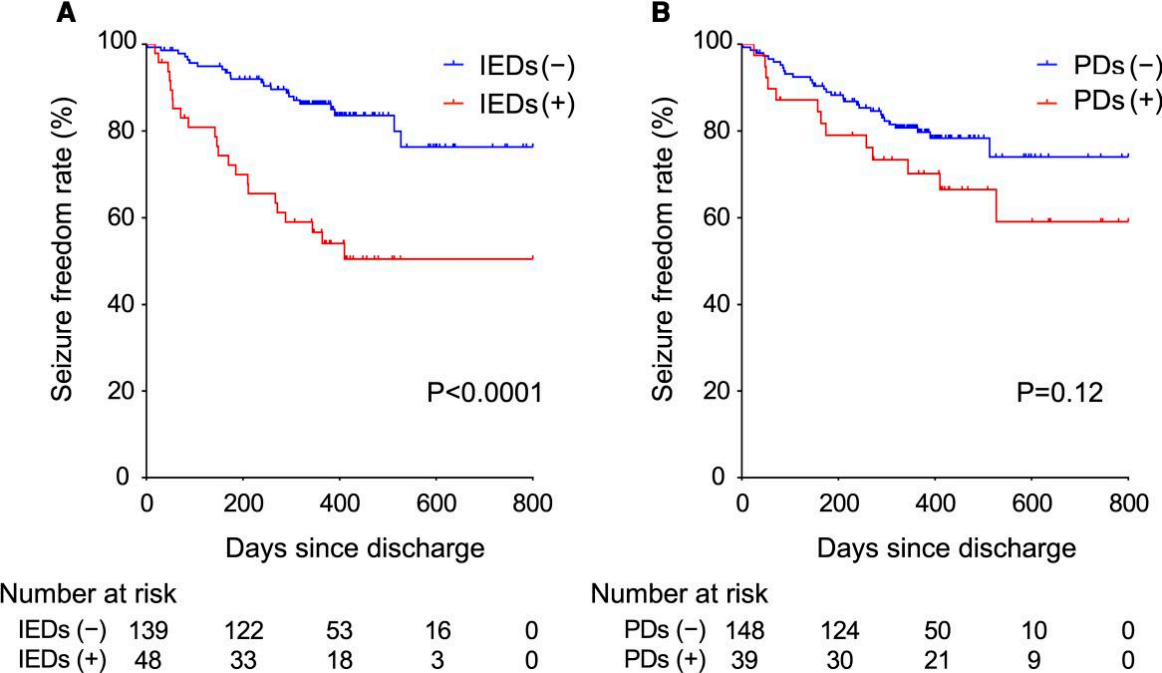
**Kaplan–Meier curves of each EEG finding for seizure recurrence.** Time to seizure recurrence according to the presence of IEDs (**A**) and PDs (**B**) in the NCVC cohort.

Among 187 patients who underwent 1 (100%) or 2 (38.0%) EEGs, IEDs, PDs, RDA and focal irregular slow were detected in 48 (25.7%), 39 (20.9%), 12 (6.4%) and 159 (85.0%) patients, respectively. The univariate Cox proportional hazards analysis showed that the IEDs group had a higher seizure recurrence risk [HR 3.82 (2.09–6.97), Table 2]. All models in the multivariable analysis demonstrated that IEDs were an independent risk factor for seizure recurrence [Model 1: HR, 3.22 (1.11–9.33); Model 2: HR, 3.36 (1.06–10.6)]. PDs, RDA and focal irregular slow were not significantly associated with seizure recurrence. Kaplan–Meier analysis indicated that IEDs were a significant risk factor for seizure recurrence, whereas the presence of PDs was not (Fig. 2).

**Table 2:** Relationship between seizure recurrence and EEG findings

	Non-seizure recurrence (*n* = 144)	Seizure recurrence (*n* = 43)	Unadjusted		Model 1^a^		Model 2^b^	
			HR (95% CI)	*P*-value	HR (95% CI)	*P*-value	HR (95% CI)	*P*-value
IEDs (*n* = 48)	26 (18.1)	22 (51.2)	3.82 (2.09–6.97)	<0.01	3.22 (1.11–9.33)	0.03	3.36 (1.06–10.6)	0.04
PDs (*n* = 39)	26 (18.1)	13 (30.2)	1.67 (0.87–3.21)	0.12	2.51 (0.82–7.67)	0.11	2.51 (0.68–9.25)	0.17
RDA (*n* = 12)	9 (6.2)	3 (7.0)	1.08 (0.33–3.48)	0.90	0 (0)	>0.99	0 (0)	>0.99
Focal irregular slow (*n* = 159)	121 (84.0)	38 (88.4)	1.41 (0.56–3.60)	0.47	1.27 (0.29–5.66)	0.75	1.06 (0.22–5.11)	0.94

Data are presented as *n* (%) or HR (95% CI).

ASMs, antiseizure medications; CI, confidence interval; HR, hazard ratio; IEDs, interictal epileptiform discharges; NIHSS, National Institutes of Health Stroke Scale; PDs, periodic discharges; RDA, rhythmic delta-wave activity.

^a^ Model 1: age, sex and NIHSS.

^b^ Model 2: Age, sex, NIHSS, haemorrhagic stroke and new-generation ASMs at discharge.

**Figure 3:**
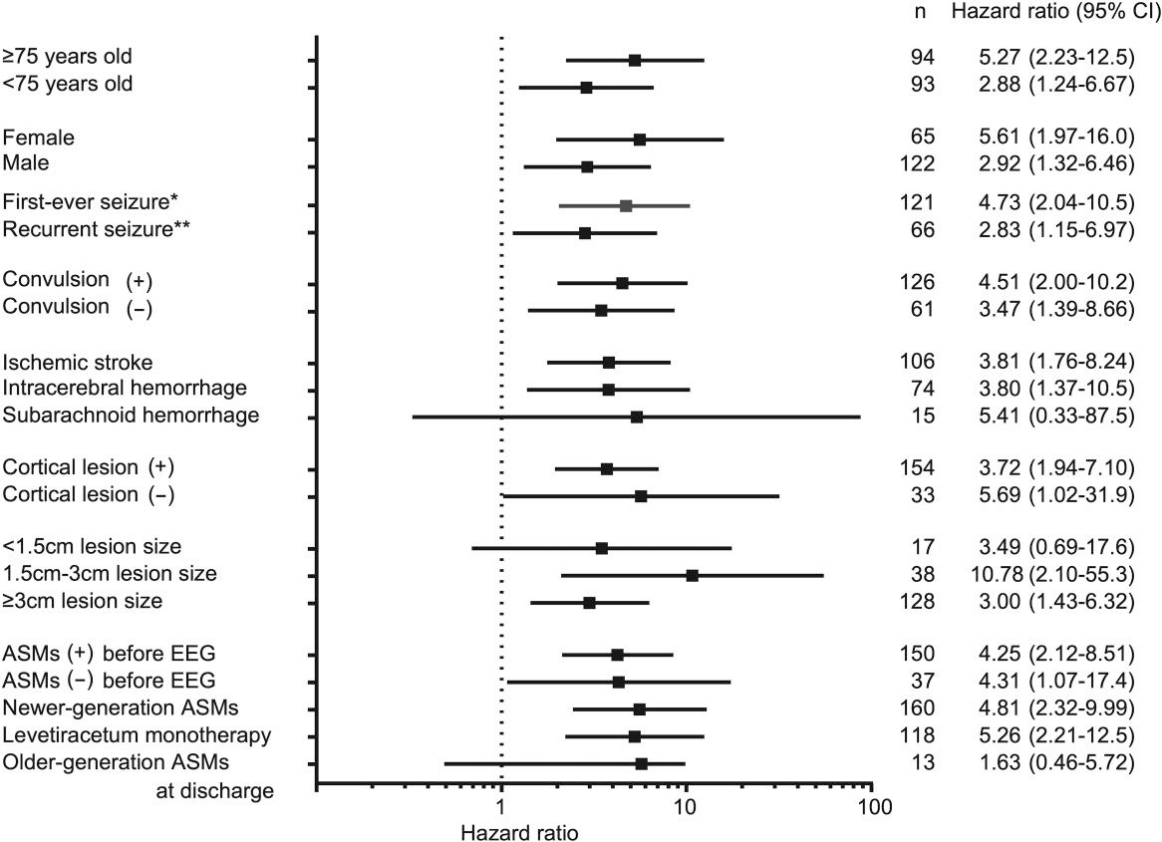
**Forest plot of HR of IEDs for seizure recurrence in the different subgroups.** The hazard risks with 95% CIs for seizure recurrence in each subgroup were calculated using a Cox proportional hazard model. *First-ever seizure refers to patients without a history of late seizures before admission. **Recurrent seizure refers to patients with a history of late seizure before admission.

With respect to the number of seizures after stroke, presence of convulsions, stroke type and lesion size, IEDs exhibited similar effect sizes for seizure recurrence (Fig. 3). Table 3 presents the relationship between EEG findings and seizure recurrence. Regardless of morphology and prevalence, IEDs were significantly associated with seizure recurrence. For PDs, the sharpness of spiky/sharp PDs was marginally associated with seizure recurrence [HR, 1.85 (0.93–3.69), *P* = 0.08].

**Table 3:** Relationship between seizure recurrence and EEG findings of IEDs and PDs

	Non-seizure recurrence (*n* = 144)	Seizure recurrence (*n* = 43)	HR (95% CI)	*P*-value
IEDs (*n* = 48)	26 (18.1)	22 (25.7)	3.82 (2.09–6.97)	<0.0001
Morphology				
Spike/spike and wave	8 (5.6)	7 (16.3)	2.58 (1.14–5.81)	0.02
Sharp/sharp and wave	18 (12.5)	15 (34.9)	3.14 (1.67–5.89)	0.0004
Prevalence				
≥1 per 10 s, but not periodic	6 (4.2)	6 (14.0)	2.72 (1.15–6.48)	0.02
≥1 per 1 min but <1 per 10 s	13 (9.0)	12 (27.9)	2.87 (1.48–5.62)	0.002
≥1 per 5 min but <1 per min	7 (4.9)	5 (11.6)	2.43 (0.95–6.17)	0.06
<1 per 5 min	8 (5.6)	8 (18.6)	2.98 (1.38–6.45)	0.006
Distribution				
Generalized	0 (0)	1 (2.3)	–	0.002
Hemispheric	3 (2.1)	2 (4.7)	2.70 (0.65–11.2)	0.17
Focal	25 (17.4)	22 (51.2)	3.96 (2.17–7.21)	<0.0001
PDs (*n* = 39)	26 (18.1)	13 (30.2)	1.67 (0.87–3.21)	0.12
Sharpness				
Spiky/sharp	18 (12.5)	11 (25.6)	1.85 (0.93–3.69)	0.08
Sharply contoured/blunt	8 (5.6)	2 (4.7)	0.93 (0.23–3.87)	0.93
Frequency				
≤1 Hz	18 (12.5)	6 (14.0)	1.00 (0.42–2.38)	>0.99
>1 Hz, but ≤2.5 Hz	4 (2.8)	3 (7.0)	2.82 (0.87–9.19)	0.08
>2.5 Hz	4 (2.8)	4 (9.3)	2.39 (0.85–6.71)	0.10
Distribution				
Generalized	0 (0)	0 (0)	–	–
Hemispheric	11 (7.7)	6 (14.0)	1.81 (0.76–4.29)	0.18
Focal	18 (12.5)	10 (23.3)	1.76 (0.87–3.58)	0.12

CI, confidence interval; HR, hazard ratio; IEDs, interictal epileptiform discharges; PDs, periodic discharges.

**Figure 4:**
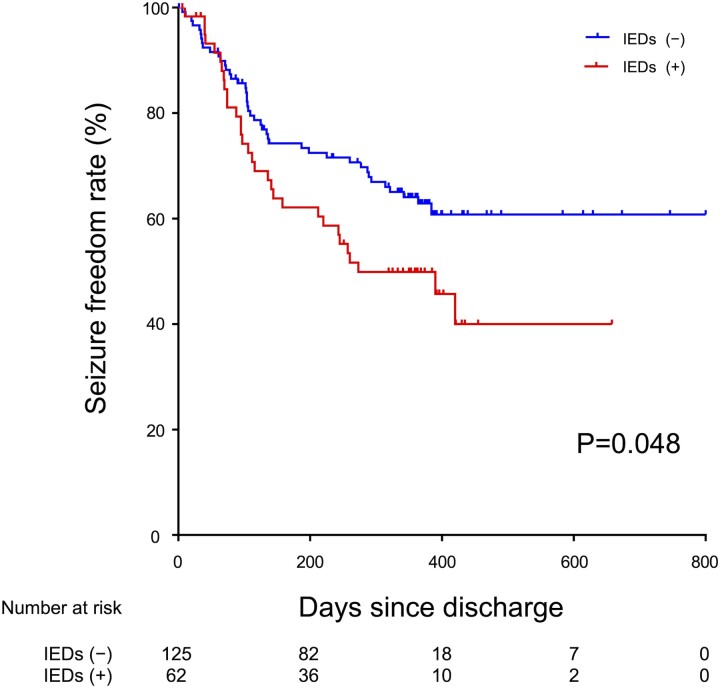
**Kaplan–Meier curves of IEDs for seizure recurrence in the validation cohort.** The seizure freedom rate at 1 year of follow-up was lower in patients with IEDs compared with non-IEDs (62.9 versus 49.9%).

In the validation cohort, 201 patients were admitted at 7 hospitals other than NCVC and 14 were excluded owing to lack of EEG data; thus, 187 patients (median age, 72 years; IQR, 64–80 years; 68 women, 36.4%) were finally enrolled (Fig. 1). Figure 4 presents Kaplan–Meier curves of IEDs for seizure recurrence in the validation cohort. IEDs were detected in 62 patients (33.2%), and seizure recurrence was observed in 31 patients (50.0%) with IEDs and 43 (34.4%) without IEDs. The seizure recurrence rate was higher in patients with IEDs (*P* = 0.048).

## Discussion

The present study demonstrated that EEG findings of IEDs indicate seizure recurrence risk in patients with PSE, regardless of prior ASMs and risk factors. The findings of the study highlight the association between seizure recurrence and EEG findings in patients with PSE. Although PDs were not statistically significant, spiky/sharp PDs were marginally associated with seizure recurrence of PSE. Furthermore, external validation analysis consistently demonstrated that IEDs were an indicator of seizure recurrence risk in patients with PSE.

Previous studies have reported that EEG abnormalities, including epileptiform abnormality and focal slowing of a background rhythm, are risk factors for seizure recurrence.^27,28^ A meta-analysis of 15 studies and 1799 participants with genetic, idiopathic, structural seizure reported that the sensitivity and specificity of IEDs in routine EEG after a single unprovoked seizure for seizure recurrence was 17.3 and 94.7%, respectively.^29^ In patients with juvenile myoclonic epilepsy, the maximum duration of epileptic discharge sequences is considered an indicator of seizure recurrence.^30^ In patients with ischaemic stroke, sharp waves/spikes on follow-up EEG after acute symptomatic seizures positively correlate with seizure recurrence risk.^23^ This research identified IEDs as a risk indicator of seizure recurrence in two PSE cohorts (both *n* = 187). A previous study reported that EEG findings did not correlate with seizure recurrence in 71 patients with PSE. However, the study differed from ours since it was a small, retrospective, single-centre study without information about ASMs regimens or detailed criteria for EEG abnormalities. Moreover, the detection rate of epileptic abnormalities was lower than that in our study (17.2 versus 25.7%).^16^ The current study may have an advantage over the previous study in that abnormal EEG findings were evaluated more extensively by board-certified epileptologists in a larger number of patients.

Additionally, spiky/sharp PDs were weakly associated with seizure recurrence. In the presence of PDs, potential spike/sharp waves can be masked by continuous periodic waves. Although few studies investigated whether the relationship between PDs and seizure recurrence exists, Bentes *et al*.^31^ reported that the presence of PDs was an independent predictor of IEDs, suggesting that spiky/sharp PDs coexist with IEDs in patients with PSE. In this regard, long-term or frequent EEG monitoring should be considered in patients with PDs.

In the subgroup analysis of the forest plot, there was no heterogeneity within each factor. Of note, the risk was similar regardless of ASMs type at discharge. There is a high degree of epileptogenesis associated with IEDs, and that can overwhelm the type of ASMs that determine seizure recurrence in PSE.^32^ A systematic review of 7082 patients among the whole epilepsy population reported that epileptiform abnormality while on ASMs was an independent predictor of seizure recurrence after ASMs withdrawal (adjusted concordance statistics, 0.65; 95% CI, 0.65–0.66).^33^ These findings suggest that the finding of EEG is an indicator of seizure recurrence, although the systematic review did not focus on PSE.

The present study had several limitations. First, there was an issue with the diagnosis of epilepsy. In particular, in the non-IEDs group, PSE was determined based solely on semiology. In this regard, 20–30% of patients diagnosed with epilepsy are misdiagnosed at epilepsy centres.^34,35^ However, in the present study, board-certified epileptologists and neurologists carefully diagnosed PSE and evaluated EEG in detail. Moreover, forest plot analysis revealed that IEDs were associated with seizure recurrence in patients with convulsions and those with two or more episodes of seizure recurrence, which strengthens the diagnostic accuracy of PSE. Second, ASMs may affect EEG findings. A systematic review including randomized controlled trials on genetically generalized epilepsy reported that ASMs reduced the density, frequency, cumulative duration and burst duration of epileptic discharge.^36,37^ In this study, the risk of IEDs for seizure recurrence was consistent regardless of the type of ASMs used before EEG. Another study limitation was the EEG recording time. Participants were examined with a routine EEG for ∼20 min, which may have been insufficient to detect low-frequency IEDs. In patients for whom diagnosis with routine EEG is difficult, long-term EEG monitoring can be useful to confirm a diagnosis.^24^ Reports indicate that prolonged EEG following a routine EEG increases additional findings by 30%.^38^ Recent studies on prolonged EEG, such as video and ambulatory EEG, reported the utility of these approaches for seizure recurrence.^39,40^ To increase the detection power of abnormal EEG findings, future studies should consider a combination of various EEGs, including long-term EEG, video monitoring and ambulatory EEG.

The strengths of this study include the following: first, the study design, which is a prospective cohort study, is less susceptible to selection and recall bias. Second, it was a multicentre study, and we were able to confirm the results with external validation. Third, several board-certified epileptologists and neurologists evaluated the EEG in detail, which may have clarified the relationship between IEDs and seizure recurrence. In this study, we also prospectively followed up on ASMs and evaluated the relationship between ASMs and EEG findings.

## Conclusion

Our findings suggest that the presence of IEDs on EEG among patients with PSE is a risk factor for seizure recurrence. Evaluating recurrence risk and treatment effectiveness using EEG may facilitate earlier intervention and thus contribute to improved prognosis.

## Supplementary Material

fcac312_Supplementary_Data

## Data Availability

All de-identified participant data generated for this work are available upon reasonable request from any qualified investigator.

## Appendix

The names of PROPOSE investigators except co-authors: Kazuyuki Nagatsuka, Fumiaki Nakamura, Shinya Tomari, Yoshitaka Yamaguchi, Takashi Nakamura, Naoki Makita, Yuki Nakamura, Yoshiaki Okuno, Satoshi Hosoki, Ryo Fujii and Takuro Arimizu.

## Abbreviations

ACNS =: American Clinical Neurophysiology Society
ASMs =: antiseizure medications
HR =: hazard ratio
IEDs =: interictal epileptiform discharges
ILAE =: International League Against Epilepsy
IQR =: interquartile range
mRS =: modified Rankin scale
NCVC =: National Cerebral and Cardiovascular Center
NIHSS =: National Institutes of Health Stroke Scale
PDs =: periodic discharges
PROPOSE =: PROgnosis of POst-Stroke Epilepsy
PSE =: poststroke epilepsy
RDA =: rhythmic delta-wave activity
