# Supplementary material for: Interictal epileptiform discharges as a predictive biomarker for recurrence of poststroke epilepsy
Source: Brain Commun. 2022 Nov 26;4(6):fcac312. doi: 10.1093/braincomms/fcac312 (PMC9746685; doi:10.1093/braincomms/fcac312)
Supplement: fcac312_Supplementary_Data [file fcac312_supplementary_data.pdf]

Supplementary Table 1. Patient characteristics with seizure recurrence

	Non-seizure recurrence (n = 144)	Seizure recurrence (n = 43)	p value
Age, years, median [IQR]	75 [66.8–82]	74 [64.5–82]	0.96
Female (%)	48 (33.3)	17 (39.5)	0.47
Family history of epilepsy	2 (1.4)	1 (2.3)	0.55
Current alcohol consumption (%)	28 (19.4)	2 (4.7)	0.02
Duration between seizure and EEG findings, days, median [IQR]	1 [1–2.8]	1 [1–2]	0.32
Comorbidities			
Hypertension (%)	119 (82.6)	38 (88.4)	0.48
Hyperlipidemia (%)	80 (55.6)	27 (62.8)	0.48
Diabetes mellitus (%)	34 (23.6)	6 (14.0)	0.21
Classification of seizures			0.20
Focal aware seizure	19 (13.2)	8 (18.6)	
Focal impaired aware seizure	52 (36.1)	16 (37.2)	
Focal-to-bilateral tonic-clonic seizure	72 (50.0)	17 (39.5)	
Others	1 (0.7)	2 (4.7)	
Ictal/postictal symptom			
Convulsion (%)	102 (70.8)	24 (55.8)	0.09
Ictal/postictal paresis (%) [a]	28 (19.9)	9 (20.9)	0.83
Aphasia (%) [a]	23 (16.3)	7 (16.3)	>0.99
Consciousness alternation (%) [a]	60 (42.6)	21 (48.8)	0.49
Stroke information			
NIHSS at the latest stroke onset [IQR]	11 [4–19]	19 [11.5–25.5]	0.02
Stroke type			
Ischaemic stroke (%)	79 (54.9)	27 (62.8)	0.39
Haemorrhagic stroke [b] (%)	70 (48.6)	19 (44.2)	0.73
Intracerebral haemorrhage (%)	57 (39.6)	17 (39.5)	>0.99
Subarachnoid haemorrhage (%)	13 (9.0)	2 (4.7)	0.53
Cortical involvement	117 (81.2)	37 (86.0)	0.65
Stroke lesion size (n=183)			0.45

<1.5 mm (%)	30 (21.3)	8 (19.0)	
15–30 mm (%)	11 (7.8)	6 (14.3)	
>30 mm (%)	100 (70.9)	28 (66.7)	
History of early seizure (%) [c]	8 (7.0)	0 (0.0)	0.20
History of late seizure (%) [c]	6 (5.3)	2 (6.5)	0.68
ASMs			
ASMs before EEG (%)	117 (81.2)	33 (76.7)	0.52
ASMs at discharge (%)	133 (92.4)	40 (93.0)	>0.99
Newer-generation ASMs [d]	113 (85.0)	23 (57.5)	0.001
Levetiracetam monotherapy	97 (72.9)	21 (52.5)	0.02
Only older-generation ASMs	3 (2.3)	10 (25.0)	<0.001
mRS at discharge, median [IQR]	3 [1–4]	3 [2–4]	0.78

Data are presented as n (%) or median [interquartile range].

Abbreviations: ASMs, antiseizure medications; IQR, interquartile range; mRS, modified Rankin scale; NIHSS, National Institutes of Health Stroke Scale.

[a] There were missing data in 3 cases, and we analyzed 184 cases.

[b] Hemorrhagic stroke includes cerebral hemorrhage and subarachnoid hemorrhage.

[c] There were missing data in 32 cases, and we analyzed 155 cases.

[d] Newer-generation ASMs include newer-generation ASMs and combination therapy of newer-generation and older-generation ASMs.

Supplementary Table 2. Antiseizure medications at discharge

ASMs n = 173			
Monotherapy (n = 140)		Polytherapy (n = 33)	
Older-generation therapy (n = 11)		Older-generation therapy (n = 2)	
CBZ	7 (4.0%)	CBZ+VPA	1 (0.6%)
VPA	4 (2.3%)	VPA+PB	1 (0.6%)
Newer-generation therapy* (n = 129)		Newer-generation therapy (n = 31)	
LEV	118 (74.6%)	LEV+LCM	2 (1.2%)
LCM	7 (4.0%)	LEV+ZNS	1 (0.6%)
ZNS	1 (0.6%)	LEV+GBP	1 (0.6%)
LTG	2 (1.2%)	LEV+TPM	3 (1.7%)
GBP	1 (0.6%)	LEV+CBZ	8 (4.6%)
		LEV+VPA	6 (3.5%)
		LEV+PHT	5 (2.9%)
		LEV+CZP	1 (0.6%)
		LTG+CBZ	1 (0.6%)
		ZNS+VPA	2 (1.2%)
		LEV+TPM+CBZ	1 (0.6%)

Abbreviations: ASM, antiseizure medication; CBZ, carbamazepine; CZP, clonazepam; GBP, gabapentin; LEV, levetiracetam; LCM, lacosamide; LTG, lamotrigine; PB, phenobarbital; PHT, phenytoin; TPM, topiramate; VPA, valproate; ZNS, zonisamide.

*Newer-generation ASMs include newer-generation ASMs and combination therapy of newer-generation and older-generation ASMs.
